# A Fatal, Post-Intubation, Tracheoesophageal Fistula

**DOI:** 10.7759/cureus.9014

**Published:** 2020-07-05

**Authors:** Sher N Baig, Stefanie J Herrera, Deborah Makinde, Fuad I Abaleka, Shahnaz Akhter

**Affiliations:** 1 Internal Medicine, Richmond University Medical Center, Staten Island, USA; 2 Research, Richmond University Medical Center, Staten Island, USA

**Keywords:** endotracheal intubation, tracheostomy, esophagoscopy, bronchoscopy, cuff, tracheo-esophageal fistula, esophageal stent, recurrent aspiration pneumonia, amyotrophic lateral sclerosis

## Abstract

Despite the use of safer tubes with high-volume, low-pressure cuffs, post-intubation injury is still the leading cause of benign, acquired, tracheoesophageal fistula (TEF). Cuff pressure, which is their primary pathogenetic driver, is not routinely monitored as a quality metric. To highlight the devastating consequences, we report this case of a fatal, iatrogenic fistula in a 64-year-old Asian male. He had undergone tracheostomy due to amyotrophic lateral sclerosis (ALS) and had a series of hospitalizations due to recurrent episodes of pneumonia. A TEF was eventually diagnosed to be the underlying cause. Esophageal stenting was ineffective. We intend to present teaching points aimed at reducing the risk of TEF in ventilator-dependent patients.

## Introduction

Post-intubation tracheoesophageal fistulas (TEFs) are rare but serious and preventable causes of morbidity and mortality in mechanically ventilated patients. Cuff pressure, which is the primary driver of the formation of a fistula, is not routinely monitored as a quality metric [1]. The result is tracheal injuries from overinflated cuffs, which account for 50%-65% of all benign, secondary TEFs [2]. Since the definitive surgical correction of TEF requires weaning off from the ventilator to allow postoperative healing of the anastomosis, it is invariably fatal in prolonged ventilator-dependent patients who cannot meet this prerequisite [3,4]. This calls for thoughtful anticipation and a multifaceted strategy to prevent the constellation of contributing factors from developing and jeopardizing the tracheoesophageal integrity in such vulnerable patients.

## Case presentation

**Figure 1:**
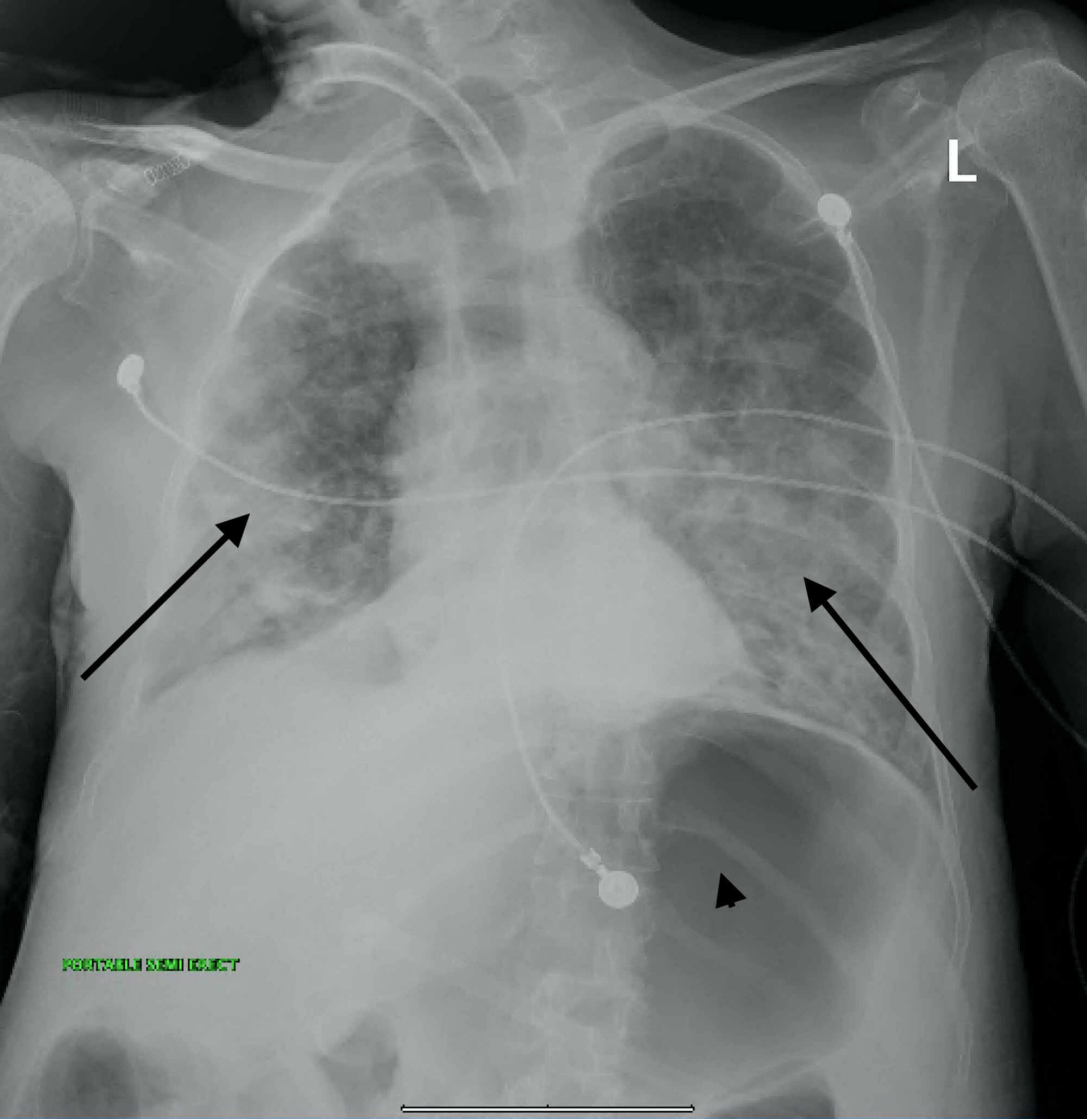
Chest radiograph Arrows point to the areas of extensive bilateral pulmonary infiltrates. Prominent gastric distention with air in the stomach is visible (arrowhead)

A 64-year-old Asian male was diagnosed with amyotrophic lateral sclerosis (ALS) 18 months before his current presentation. His other medical problems included hypertension, diabetes, and epilepsy. He had undergone tracheostomy and percutaneous endoscopic gastrostomy (PEG) placement in the aftermath of his diagnosis for supportive care. In the past few months, he had been hospitalized several times to different New York City hospitals with respiratory distress, fever, cough, regurgitation, and incessant tracheal secretions. He had been treated serially for recurring pneumonia. On recent admission to a different hospital one-month ago, he was evaluated with an esophagoscopy and a barium swallow study but no evidence of TEF was found and the cause of refractory pneumonia remained unknown. He presented to our hospital with increased ventilator requirements, sepsis, and copious secretions from the tracheostomy tube. Extensive bilateral pneumonia and gaseous gastric distension were seen on a chest x-ray (Figure 1).

He was otherwise alert, oriented, and responsive. Sputum culture was notable for Acinetobacter growth. His clinical course was waxing and waning with only partial response to antibiotic regimens.

**Figure 2:**
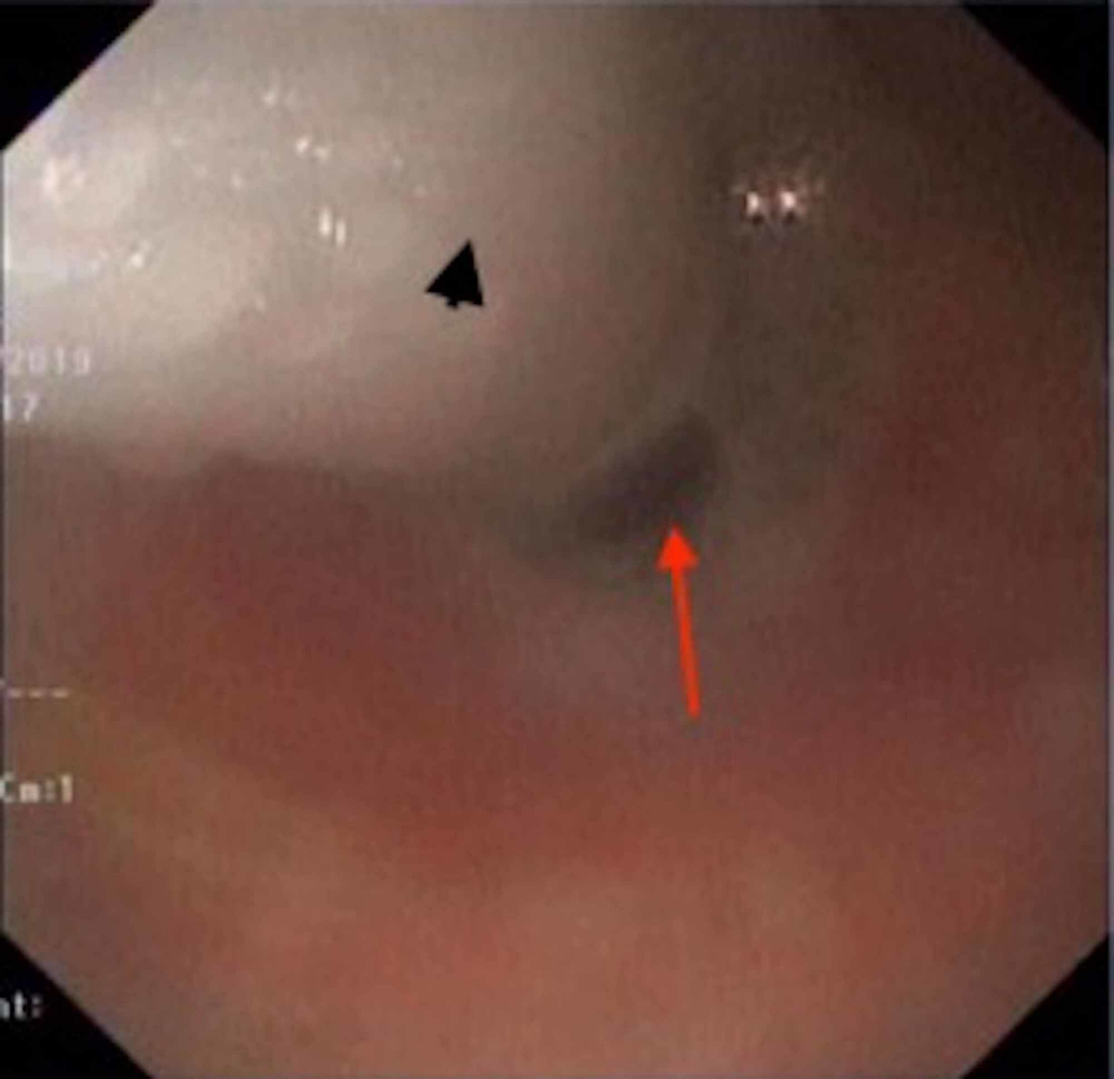
Esophagoscopic view The inflated cuff of tracheostomy tube (black arrowhead) is seen inside the esophagus. The red arrow points to the gap beside the cuff due to the large fistula size of the fistula

The gastroenterology team was called to help elucidate his falling hemoglobin level. After an initial abnormal EGD suspicious for TEF, a multidisciplinary team was made up of a gastroenterologist, an intensivist, and a thoracic surgeon to conduct a detailed examination of his aero-digestive tract and endoscopic intervention. Simultaneous esophago-bronchoscopy showed the inflated balloon of the tracheostomy tube eroding into the esophagus (Figure 2).

**Figure 3:**
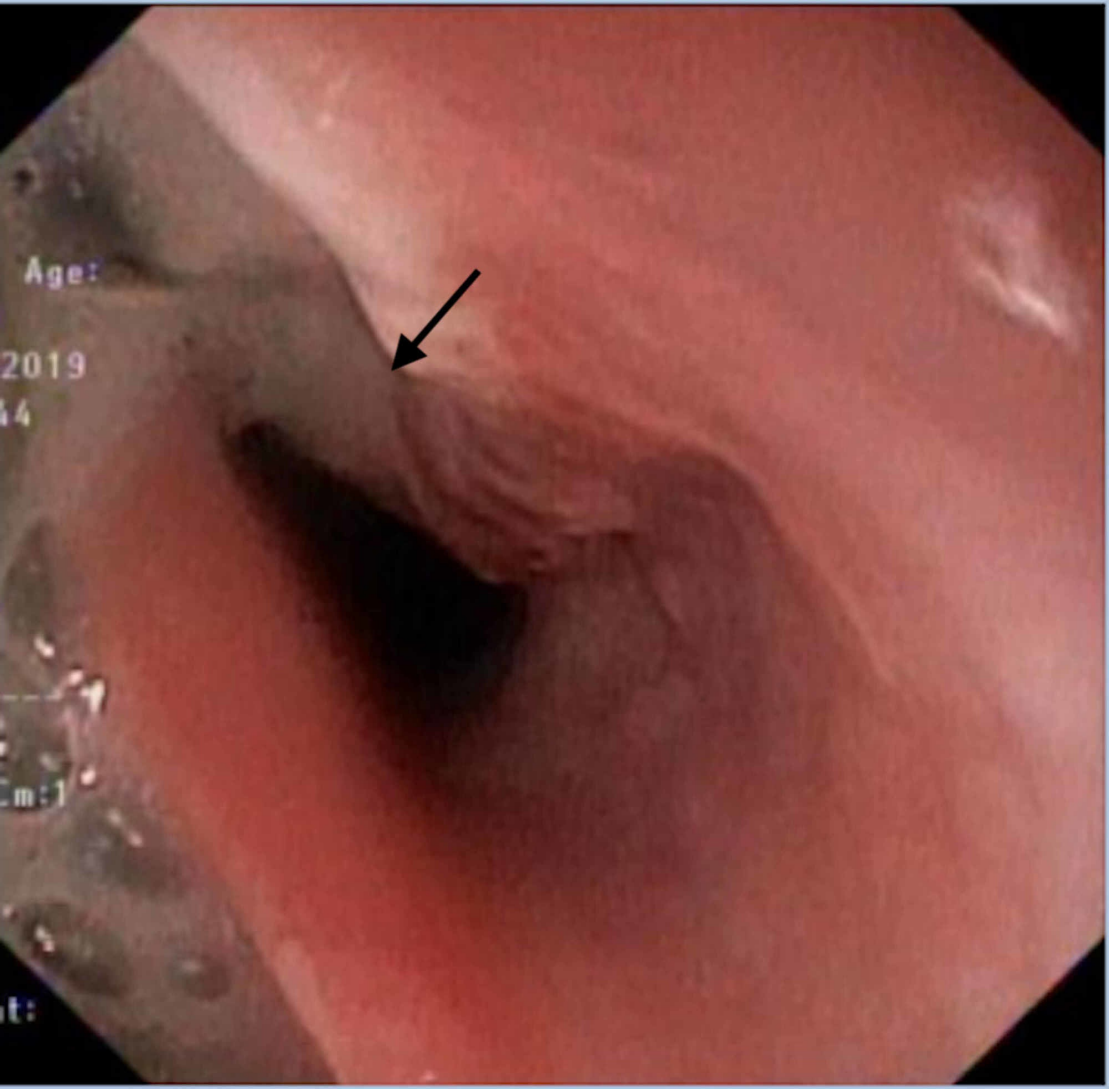
The cuff has been deflated and a large fistula is visible with tracheal secretions entering the esophagus (arrow)

On deflating it, a large TEF approximately 5 cm in length was identified 15 cm from the incisors (Figure 3). It partly involved the upper esophageal sphincter (UES). Bronchoscopy revealed a large (4.8 cm) upper tracheal defect, and moderate to severe tracheobronchitis with suppuration.

**Figure 4:**
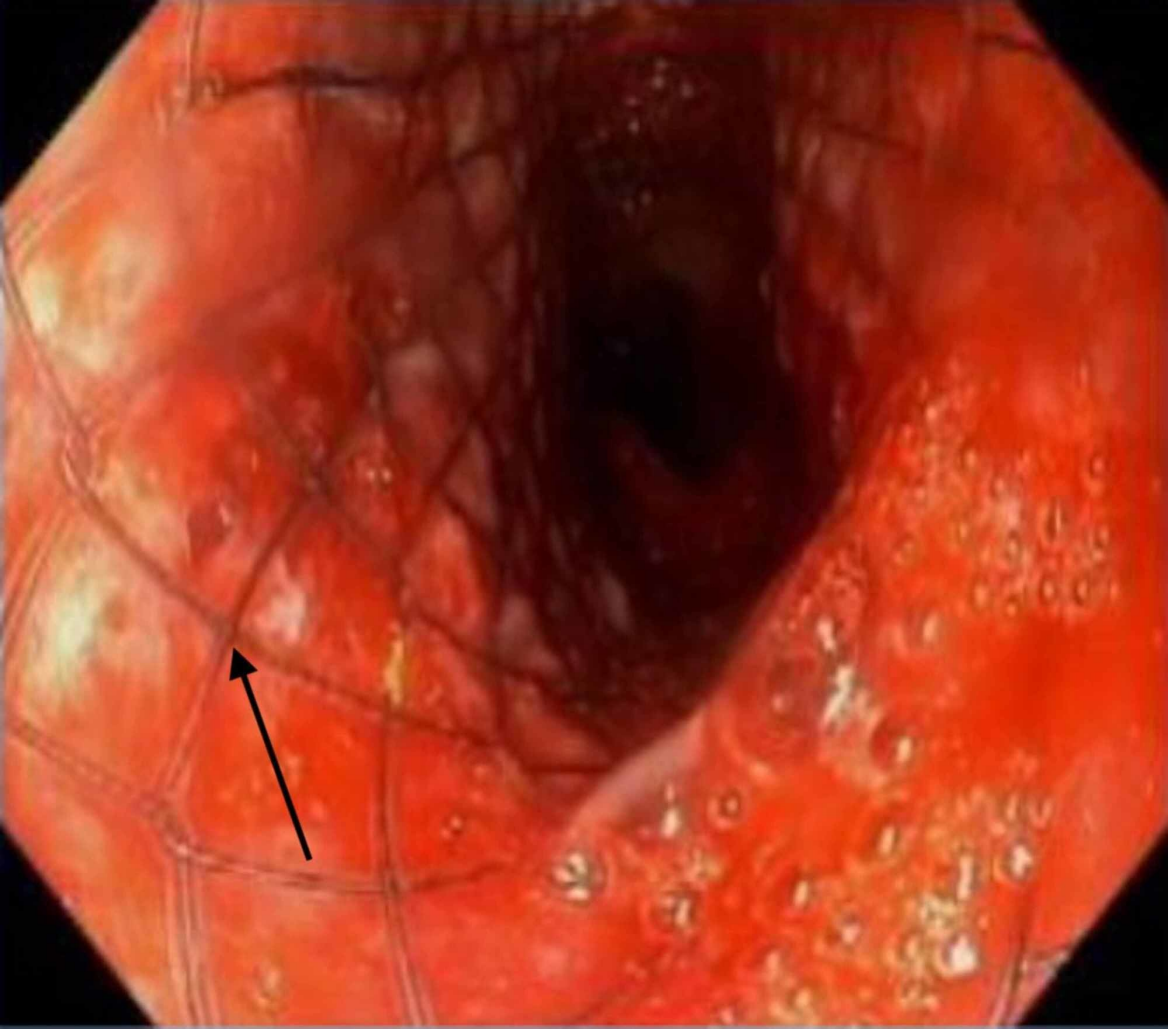
A covered metallic endoluminal esophageal stent has been deployed to seal the defect

A 22 mm x 8 cm fully covered metallic esophageal stent was deployed (with the proximal end within <1 cm of the UES) under fluoroscopic guidance to seal the defect (Figure 4).

A longer tracheostomy tube was positioned distal to the tracheal defect bronchoscopically. In the days following the successful stenting, air leak from the trach tube stopped, leukocytosis resolved, and the volume of tracheal secretions decreased. The patient also started to tolerate PEG feeding. On day 4, however, the feeds began to resurface in the tracheostomy, so feeding was stopped and total parenteral nutrition (TPN) was initiated. On repeat esophagoscopy, the stent had somewhat migrated downwards partially exposing the defect at the proximal end, leading to its failure. The critical location of the fistula encroaching the UES and its unusually large size were both bad prognosticators of stent failure. Due to the debilitating neurologic state, the patient and his family did not want any further surgical interventions. His mental and hemodynamic status inexorably deteriorated until he expired in a month.

## Discussion

Most TEFs are traumatic in origin due to prolonged mechanical ventilation, surgery, chest injury, or infection. The formation of a TEF in a mechanically ventilated patient is very rare but has serious implications for morbidity and mortality. With the disuse of high-pressure cuffs in favor of high-volume, low-pressure cuffs, the overall incidence of tracheal injuries has decreased. TEFs are estimated to occur in 0.5% to 1% of prolonged ventilated patients [1]. The most prevalent cause (47%) of benign, acquired, TEF is post-intubation injury [2]. The high cuff pressure is a strong risk factor [4,5]. The cuff seals against the tracheal wall to prevent air leakage and ensure the delivery of tidal volume to the lungs. When cuff pressure exceeds >30 cm H2O it compresses mucosal capillaries and impairs blood flow with tracheal tissue ischemia and necrosis eventually [5]. Prolonged duration of mechanical ventilation, direct mechanical trauma from manipulation of the tube during dressing, and respiratory care are other predisposing factors [5]. Systemic factors such as hypotension, shock, hypoxemia, anemia, and metabolic acidosis also increase the risk [5]. The presence of both nasogastric and endotracheal tubes also increases the risk of mechanical injury to the interposed tracheoesophageal walls [5].

A high index of suspicion is required for diagnosis as most patients are intubated and non-verbal at the time. TEF should be suspected in a ventilated patient if there is a constant air leak, recurrent pneumonia, respiratory distress, presence of enteral feed in tracheal aspirate during suctioning, and gastric distension. In an extubated patient, coughing after swallowing may raise suspicion for a TEF [5]. TEFs can be visualized via EGD and/or bronchoscopy. Smaller defects can be investigated by barium esophagram. Once the diagnosis is confirmed, the cuff position should be adjusted distally to the fistula to minimize pulmonary soiling [3]. Frequent, gentle suctioning and elevation of the head of the bed help to reduce contamination. A gastrostomy tube should be inserted and kept on drainage to reduce the reflux of gastric contents [3,4]. An esophageal stent also limits aspiration and may allow swallowing in selected patients. NGT should be removed and the jejunostomy tube should be placed for nutrition [3]. Some authors favor TPN instead.

The definitive treatment of post-intubation TEF is surgical. Due to the danger of tracheal anastomotic breakdown from positive pressure mechanical ventilation surgical correction is performed after the patient is liberated from the ventilator [1,3,4]. This prerequisite excludes most ventilator-dependent like this patient from surgery. In otherwise eligible patients, successful closure can be achieved with several surgical techniques with the restoration of normal breathing and swallowing [2]. The choice of the procedure varies based on size, location, and etiology. Extra-thoracic TEF requires cervicotomy, while an intra-thoracic TEF requires right thoracotomy. The low anterior cervical collar approach is the most popular technique with a success rate of 95% [6]. Tracheal or esophageal stents may have a limited temporizing role [4]. Dual esophageal and tracheal stenting can be considered if there is airway stenosis, or TEF is >2 cm or proximally located. Tracheal stenting alone is an option if an esophageal stent is not indicated or is unable to be placed [7]. Some reports have shown successful endoscopic closures of small TEFs (<5 mm in size) with over-the-scope-clipping (OTSC) [8]. Without prompt palliation, death occurs within six weeks [3].

Due to the poor outcomes associated with TEF in ventilator-dependent patients, it is essential to take preventive steps. Early extubation protocols should be followed. Deep suctioning should be avoided. Cuff volumes and pressures should be closely monitored and incorporated into the routine care of ventilated patients as a quality metric. Some newer tubes with tight-to-shaft (TTS) cuffs eliminate the lip that forms when standard cuffs are deflated and may offer better safety for long-term use.

## Conclusions

Recurring pneumonia in intubated patients should raise the suspicion of a TEF. This is a deadly complication of prolonged mechanical ventilation, especially since the surgical correction is not feasible in ventilator-dependent patients. By monitoring cuff pressures in routine clinical practice to ensure lower cuff pressures, especially in long-term ventilator-dependent patients, the risk of post-intubation tracheoesophageal fistulas may be reduced.
